# The identification of diurnal variations on circulating immune cells by finger prick blood sampling in small sample sizes: a pilot study

**DOI:** 10.1093/labmed/lmad062

**Published:** 2023-07-21

**Authors:** Dayna Bushell, Jonathan Kah Huat Tan, Jessica Smith, Christian Moro

**Affiliations:** Faculty of Health Sciences and Medicine, Bond University, Robina, Australia; Clem Jones Centre for Regenerative Medicine, Bond University, Robina, Australia; Clem Jones Centre for Regenerative Medicine, Bond University, Robina, Australia; Faculty of Health Sciences and Medicine, Bond University, Robina, Australia

**Keywords:** T cells, variations, blood, lymphocytes, physiology, clinical

## Abstract

**Objective:**

There are well-described impacts of biological rhythms on human physiology. With the increasing push for routine blood tests for preventative medical care and clinical and physiological research, optimizing effectiveness is paramount. This study aimed to determine whether it is feasible to assess diurnal variations of peripheral lymphocyte prevalence using finger prick blood in a small sample size.

**Methods:**

Using polychromatic flow cytometry, the prevalence of lymphocytes was assessed using 25 µL fingertip blood samples at 8 AM and 5 PM from 8 participants.

**Results:**

TH cells and B cells showed significantly higher percentages in the 5 PM samples, whereas NK cells demonstrated a significantly higher morning percentage. T cells, leukocytes, and cytotoxic T cells showed no significant changes.

**Conclusion:**

The detection of diurnal variations demonstrates that small blood volumes can be used to detect lymphocyte variations. The lower blood volume required provides a new testing method for clinical and research settings.

## Introduction

### Diurnal Variation

Many physiological events, including immune responsiveness, are linked to the circadian rhythm. Diurnal changes are a form of biological rhythms that show variations synchronized with the time of day.^1,2^ Diurnal variation is observed across many areas in human physiology and immunology, including hormone secretion and heart rate.^3-7^ In addition to physiological responses, diurnal variation can also be observed in individual cells. Circulating blood leukocyte counts have demonstrated peak numbers in humans, mice, rats, and hamsters during the behavioral rest phases.^8-12^ The diurnal pattern of leukocytes in murine models has been linked to the egress of cells from hematopoietic organs into the blood and the immigration of cells to peripheral organs.^9,13^ The same molecular mechanism for the cell-intrinsic clock is present in both innate and adaptive murine, rat, and human immune cells.^14-20^

### Volume of Blood Commonly Used in the Assessments of Physiological Diurnal Variation

Previous studies of physiological lymphocyte diurnal variation have predominantly used venipuncture to assess cell prevalence. An early study by Miyawaki et al^21^ used 2 mL of blood drawn via syringe from the medial cubital vein for each timepoint. More recent studies by Boukelia et al^22^ and Beam et al^23^ used similar volumes of 8 mL and 5.5 mL, respectively, drawn using venipuncture. The studies by Lévi et al^24^ and Mazzoccoli et al^25^ used larger venous blood draws of 40 mL and over 100 mL, respectively. Other studies have not specified the volume drawn and used but have stated that venipuncture was used to collect peripheral blood samples.^26,27^

### Blood Testing

There is an increasing use of blood testing in medical practice, extending beyond solely diagnosis, with emphasis also on preventative medicine. In normal practice, a relatively large volume of blood is drawn (usually >10 mL). However, modern machines and testing equipment have become so precise that volumes in the microliters are all that is required for widespread assessments. This means future blood testing may draw far less blood, making tests faster and with less impact on patients. In addition, small volumes of blood can be assessed from finger-prick, from the ear, or other accessible points on the patient’s extremities.^28^ This will be less painful and a safer and easier process than the normal use of venipuncture. Furthermore, there is a possibility that time of day may affect the interpretation of peripheral lymphocytic assessments, as the prevalence of lymphocytes may vary in peripheral blood. Although both lymphocyte detection in small blood samples from large sample sizes^29^ and lymphocyte diurnal variation have been established, it has not been determined whether significant variations in lymphocyte prevalence can be detected through small blood volume samples in a small sample size. As such, by determining whether a variation can be detected using small, finger prick blood samples, the methodology for clinical blood tests and physiological research can be improved, leading to better participant acceptability, a less invasive sampling technique, and an increased understanding of normal variations in lymphocytes.

### Study Rationale

The time of day is often a consideration in both human population biology research and clinical assessments of allergies, autoimmune diseases, and immune-mediated inflammatory issues. However, when coupled with physiological analyses, this is often performed by drawing relatively larger volumes of serum and blood than required by the testing equipment and methods. The aim of this study was to assess whether this diurnal variation can be detected in small-volume blood draws from a small sample size and whether this is a potential future technique for the assessment of immune cells. The identification of these variations in small, finger-prick blood volumes will expand on the current literature surrounding lymphocyte diurnal variations and provide information on whether they are an important factor in finger-prick blood collections in both clinical practice and laboratory research. The outcome will be whether it is feasible for a finger-prick volume of blood to be taken and assessed in place of typical venipuncture volumes, leading to improvements in blood draw methodology and contributing to the current research into physiological diurnal variation.

## Materials and Methods

Participants were recruited from a local university and all aged between 18 and 25 years. Eight healthy volunteers participated (5 males and 3 females), with blood samples being taken from each person at 2 time points, 8 AM and 5 PM, on the same day. Unlike red blood cell testing, pathology laboratories and blood test reference ranges do not take the patient’s sex into account for the assessment of lymphocytes.^30^ As such, males and females were grouped in this study. This number of subjects and the practice of merging sexes for white blood cell analyses are also commensurate with similar recent research in this area.^22,23,31^

Participants were not required to fast. During the collection procedure, participants had warm water applied to their fingertips to increase localized blood flow, and this was allowed to dry. A minimum of 225 µL of finger-prick blood was collected using safety lancets into two 200-µL Microvette, K3 EDTA tubes (Sarstedt Group). When possible, all collection was completed using a single fingertip. An IMK Simultest: Lymphocyte Kit (Becton Dickinson) was used for analysis with a methodology adapted from the relevant manual volume (BD Biosciences) to account for the reduced blood. Although other reagents are available, this kit is a commonly used and commercially available kit, with a long shelf life and ready-to-use reagents to conduct lymphocyte assessments. TABLE 1 outlines the reagents provided with the kit. Additionally, 2 single stain controls were used, CD11a fluorescein isothiocyanate (FITC) mouse anti-human antibody (Ex_max_ 494 nm/Em_max_ 520 nm) and CD86 phycoerythrin (PE) mouse anti-human antibody (Ex_max_ 496 nm/Em_max_ 578 nm). To account for the lower blood volume, all volumes stated in the manual were divided by 4, resulting in 25 µL blood and 5 µL reagent. Following conjugate antibody staining for 20 minutes at room temperature, red blood cells were lysed for 10 minutes at room temperature using 500 µL 10x red blood cell lysing solution. After centrifugation (4°C at 200*g*) and discarding the supernatant, the samples were washed with 500 µL phosphate buffered saline (PBS) (Sigma Life Sciences). Following centrifugation and discarding the supernatant, samples were resuspended in 125 µL solution (PBS without calcium, chloride, or magnesium, with 2% fetal bovine serum and 0.1% sodium azide). All samples, excluding unstained, and FITC and PE were mixed with 2 µL of propidium iodide.

**TABLE 1.** Summary of reagents from the IMK Simultest Kit

Reagent	Specificity	Clone	Conjugation	Cell ID	Concentration (µg/mL)
A	CD45	2D1	FITC	Leucocytes	2.5
	CD14	M*φ*P9	PE	Monocytes	12.5
B	IgG1	X40	FITC	Nonspecific Fc receptors	12.5
	IgG2a	X39	PE	Nonspecific Fc receptors	12.5
C	CD3	SK7	FITC	CD3/TCR	25
	CD19	4G7	PE	B cell	3.1
D	CD3	SK7	FITC	CD3/TCR	25
	CD4	SK3	PE	Helper T4 cell	1.5
E	CD3	SK7	FITC	CD3/TCR	25
	CD8	SK1	PE	Cytotoxic T8 cell	6.25
F	CD3	SK7	FITC	CD3/TCR	25
	CD16	B73.1	PE	NK cell and neutrophil	6.25
	CD56	MY31	PE	All NK cells and 5% of CD3 cells	12.5

FITC, fluorescein isothiocyanate; NK, natural killer; PE, phycoerythrin.

### Flow Cytometry

Samples were analyzed on a BD FACSVerse cytometer (Becton Dickinson). Gating and compensation were conducted using FlowJo v10.8.1 software (Becton Dickinson). Lymphocyte gating was conducted individually for each participant with forward scatter-area (FSC-A)/side scatter-area (SSC-A) parameters. Propidium iodide live/dead cell gating using propidium iodide conjugated with peridinin chlorophyll protein-cyanine5.5 (PerCp-Cy5.5) (Ex_max_ 482 nm/Em_max_ 695 nm) was next performed with FSC-A/ PerCp-Cy5.5 parameters. Finally, singlet gating was conducted using sequential forward scatter-height/forward scatter-width and side scatter-height/side scatter-width gates. Samples were then analyzed using FITC and PE quadrants to identify surface markers and cell type.

### Statistics

Data collected from this study was analyzed using Prism v9 (GraphPad Software). A paired Student 2-tailed *t-*test was used to analyze the morning and afternoon lymphocyte prevalence counts. A *P* value of less than .05 was considered significant.

### Ethics

Ethics was approved by the university human research ethics committee. A written explanatory statement was provided to all participants involved, outlining the details of the project, methods of data collection, analysis confidentiality and the contact details of the study supervisor for any potential queries. Written informed consent was also obtained from all participants before the collection of the morning blood sample.

## Results

**Figure 1.**
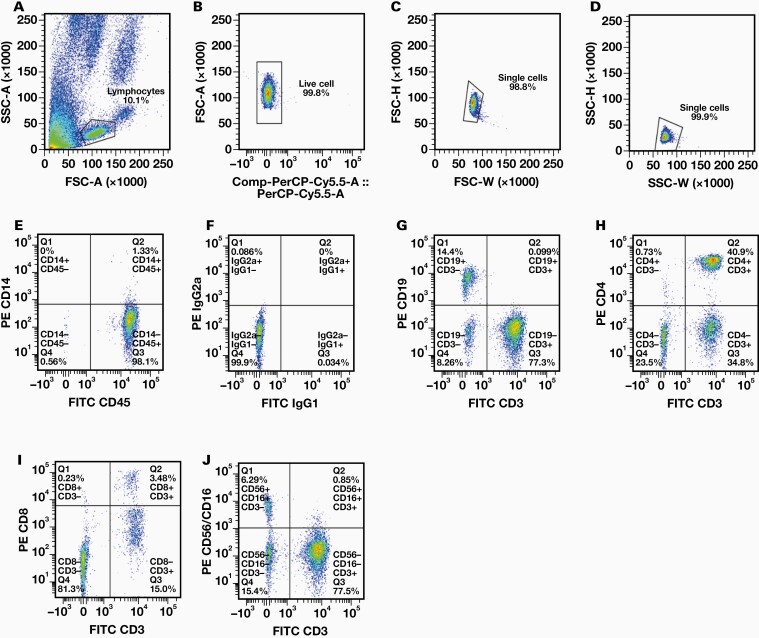
Gating method for samples. A, Lymphocytes are first selected based on forward scatter area (FSC-A) and side scatter area (SSC-A) parameters. B, Peridinin chlorophyll protein-cyanine5.5 (PerCp-Cy5.5) vs FSC-A dot plot showing gating for live cells following lymphocyte gating. Singlet gating is performed using FSC-weight (W) vs FSC-height (H) dot plots (C) and SSC-W vs SSC-H dot plots (D). E-J, Cell staining examples using fluorescein isothiocyanate (FITC) and phycoerythrin (PE) conjugated antibodies for one participant following lymphocyte, live cell, and singlet gating.

Both volumes of blood, 100 µL and 25 µL were processed by the flow cytometer without error. FIGURE 1 demonstrates an example of the gating technique used for a 25 µL sample.

### Assessment of Low-Volume (25 µL/Reagent) Blood Compared With Standard Practice (100 µL/Reagent)

An initial assessment using a single volunteer was completed to confirm the viability of using a smaller volume of blood based on the premise outlined in the study by Moro et al.^28^ Both 100 µL and 25 µL of blood reported similar results within the reference ranges (TABLE 2). As such, from this point, the smaller volume of blood was used to assess diurnal variation.

**TABLE 2.** Comparison reference ranges (BD Biosciences IMK Simultest Lymphocyte manual) and original (100 µL) vs modified (25 µL) methodologies as percentages of total gated lymphocytes^a^

	100 µL	25 µL	95% Reference range
CD3+ (T) cell, %	62.1	60.9	72 (59-85)
CD3^+^CD4^+^ (T-helper) cell, %	35.1	35.1	46 (31-61)
CD3^+^CD8^+^ (cytotoxic) T cell, %	29.7	23.8	25 (11-38)
CD4^+^/CD8^+^ cell ratio, %	1.8	1.5	1.9 (0.9-3.6)
CD19^+^ (B) cell, %	8.59	10.6	13 (6.4-23)
CD16^+^ 56^+^ (NK) cell, %	9.26	6.61	14 (5.6-31)

^a^Samples taken from 1 participant. Reference ranges obtained from BD Biosciences IMK manual.

### Assessment of Diurnal Variation

**Figure 2.**
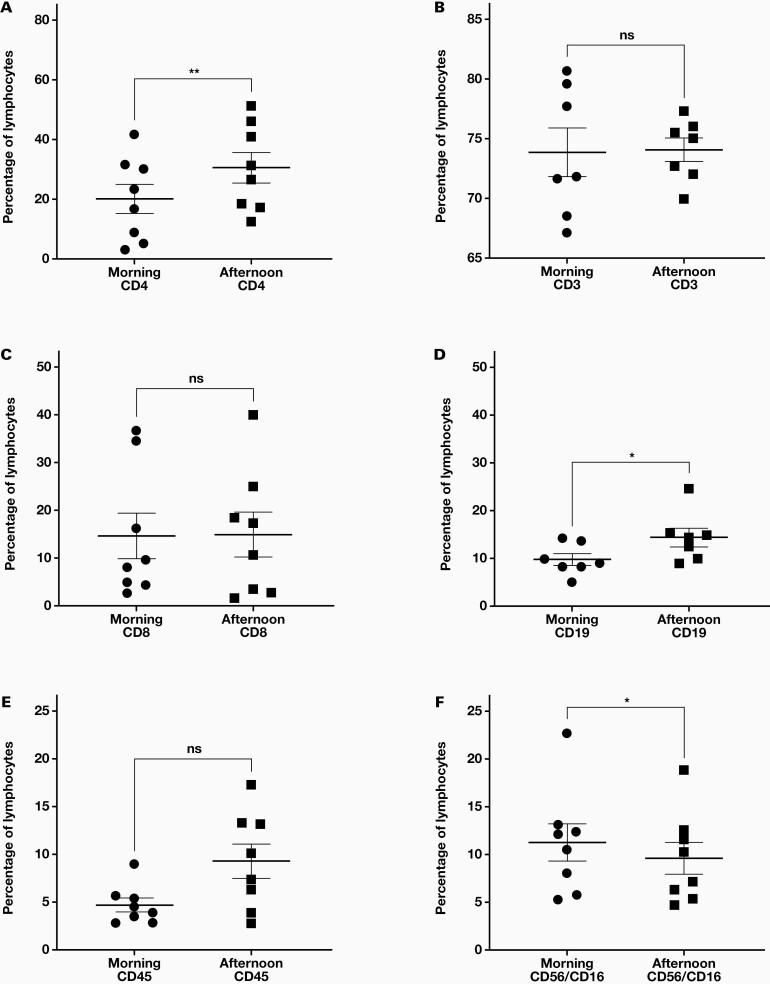
Individual values and mean ± SEM (line) for morning (9 AM) and afternoon (5 PM) blood samples (A, C, E, F: n = 8; B, D: n = 7). ns, not-significant, *P < .05, ** *P* < .005.

Helper T cells (CD4+) showed a significantly (*P* = .0023) higher percentage in the afternoon sample, with a mean difference between the morning and afternoon samples of 10.48% (n = 8, FIGURE 2). B cells (CD19+) showed a significantly (*P* = .0123) higher percentage in the afternoon sample with a mean difference of 4.62% (n = 7). NK cells (CD56+/CD16+) showed a significantly higher percentage in the morning (*P* = .0462) with a mean difference of 1.64% (n = 8). Lymphocytes (CD3+) showed no significant difference between samples (n = 8). T cells (CD3+) (n = 7) and cytotoxic T cells (CD8+) (n = 8) also showed no significant changes.

## Discussion

### Feasibility and Use of Low-Volume Blood Collection Methods

Typical assessment using a BD Biosciences IMK Simultest Lymphocyte Kit use 100 µL blood per reagent, totaling a minimum collection of 900 µL, excluding potential clotting, in comparison to the adapted methodology of 25 µL per reagent, totaling a minimum of 225 µL. Collecting over 900 µL of blood is not viable from a finger-prick, requiring venipuncture. In comparison, 225 µL blood can be collected from a fingertip blood sample before clotting occurs. Although this study investigated the effect of diurnal variation on lymphocytes, the successful use of fingertip blood to assess the prevalence and variation of these cells demonstrates a potential new technique for peripheral lymphocytic assessments. Furthermore, this study also demonstrates that this technique is feasible to detect significant variations in lymphocyte prevalence with a small sample size. Although previous studies are largely dated and present varying results, the significant variations detected do correlate with some previous results with 2 circadian assessments determining significant B-cell peaks in the afternoon and evening.^23,31^ However, 1 of these studies attributed the changes to individual variations, rather than circadian variation,^23^ requiring further research to establish the effect of diurnal variation on B-cell levels. Five of 7 studies also demonstrated that helper T cells predominantly display peaks in the afternoon and late evening.^23,31-33^ The lack of significance present in T cells, cytotoxic T cells, and general lymphocyte levels may occur due to small sample sizes and as such, further research is needed to determine whether these variations can be detected accurately using this method. This demonstrates that this technique can accurately detect daily variations in B cells and helper T cells, in line with the literature, within a small sample size.

For similar applications in science and medicine, where blood draws from small groups are required, this technique can be used to identify variations in lymphocyte counts. For people adverse to venipuncture, the use of finger-prick blood sampling may present a less invasive method of analyzing immune cell levels. In addition to general fears of venipuncture, there are several reported complications with this method,^34^ meaning that the use of low-volume blood collection methods presents a safer technique for blood collections, especially in situations where a person is having to undergo frequent or routine blood draws. Additionally, there are many groups of the population where venipuncture is not appropriate. For example, people who put regular strain on their arms, such as kayakers and weightlifters, may not be able to use venipuncture and as such finger-prick blood collections for immunological assessments may be far more appropriate.^28^ Although there are limited opportunities for venipuncture around the body, the need to only require 25 µL of blood for each cell of interest opens far more locations for blood extraction in the body, including from fingertips, toes, and ears. Therefore, the ability for lower volumes of blood to conduct peripheral lymphocytic assessments, even in limited sample sizes, provides a new, minimally invasive method which can be used in future studies of peripheral immunological assessments or in clinical settings where blood tests are commonplace.

### Potential Importance of Diurnal Variation

Although it is unclear whether diurnal variation of lymphocytes presents any biological significance, diurnal variation is a methodological problem in both population biology and clinical biology. In population biology, daily variations of immune cells can lead to variances in results, which do not relate to the topic being studied. The significant variations seen in this study from a small sample size also highlight potential impacts on some clinical tests, such as those that analyze lymphocyte prevalence to monitor HIV and lymphoma progression and treatment. As such, blood draws in a clinical setting should consider time of day in these assessments to ensure accurate interpretation of peripheral lymphocyte results. The immunological roles of lymphocytes may correspond with the variations that were observed. The increase of helper T cells, specifically regulatory T cells, and B cells in the afternoon may be due to their regulatory and anti-inflammatory function, with their mechanisms enhancing the environment to allow for tissue repair and regeneration during the nighttime rest phase.^35,36^

In clinical settings, the time-dependent changes of lymphocyte subsets observed may be responsible for the variation of immune responses in autoimmune diseases, allergies, and immune inflammatory conditions. Although diurnal variation in autoimmune disease symptoms has been reported,^37-39^ there is still a lack of clarity regarding the link between symptom presentation and cell variation. Due to the wide range of immune cell and lymphocyte subsets and their intertwining roles, it is currently not possible to fully establish the effect of immune diurnal variation autoimmune disease symptoms. However, it is possible that a dysregulation or disruption of circadian rhythms may lead to the presence of autoimmune disease symptoms by creating an inflammatory environment due to abnormal immune cell levels.^40^ Additionally, the decreased B-cell levels in the morning sample may correspond with increased reporting of autoimmune disease allergic and asthmatic symptoms in the morning. In particular, as regulatory B cells regulate the immune response and restrict excessive inflammation, a decrease of these cells may correlate with the increased morning symptoms of immune conditions related to excessive inflammation.^41^

### Limitations

This study used a small sample size, which can limit the applicability of statistical significance measured. Due to the small sample size, differences from individual factors can be shown that may not be fully attributable to diurnal variation. Additionally, when comparing with previous studies that have analyzed diurnal variation to corroborate results, most of the studies are dated and display varied times for peak lymphocyte levels. As such, future research is needed with a second cohort and larger sample sizes to further establish whether significant diurnal variations can be accurately detected when using small blood volumes.

## Conclusions

Although previous studies have shown that lymphocytes can be detected in small blood samples from large sample sizes and that lymphocyte diurnal variation occurs, this study demonstrates that significant daily variations in lymphocyte counts can be determined in a small sample size from a small volume fingertip blood sample. Although it is unclear in the literature whether this variation is physiologically significant, it is possible that the physiological immune roles of the lymphocytes link to their functions. The effective use of a small fingertip blood volume in a limited sample size to detect these variations demonstrates that this technique can be applied in both physiological research, including lymphocytes and clinical practice during blood tests to identify variations present in lymphocyte prevalence. The use of this technique presents a less invasive and more acceptable technique for participants and patients. This study demonstrates small fingertip blood volumes, even when taken from small sample sizes, can detect significant lymphocyte diurnal variation, with this influencing future methodologies and considerations in clinical practice in addition to physiological and immunological research settings.

## Abbreviations

FITC: fluorescein isothiocyanate
PBS: phosphate buffered saline
PerCp-Cy5.5: peridinin chlorophyll protein-cyanine5.5
PE: phycoerythrin
